# Erratum to: Does a tailored, multilevel implementation programme help clinicians to apply shared decision-making in breast cancer care? A before-after study

**DOI:** 10.1093/bjsopen/zrab008

**Published:** 2021-05-14

**Authors:** H van Veenendaal, H R Voogdt-Pruis, D T Ubbink, C G J M Hilders

**Affiliations:** 1 Erasmus School of Health Policy and Management, Erasmus University Rotterdam, Rotterdam, the Netherlands; 2 Dutch Association of Oncology Patient Organizations, Utrecht, the Netherlands; 3 Department of Global Health, University Medical Centre Utrecht, Utrecht, the Netherlands; 4 Department of Surgery, Amsterdam University Medical Centre, Amsterdam, the Netherlands; 5 Reinier de Graaf Hospital, Delft, the Netherlands

*BJS Open*, doi: 10.1093/bjsopen/zraa002

In the originally published version of this manuscript, requested author amendments were inadvertently omitted prior to publishing, as noted and listed in this erratum.

Co-author H.R. Voogdt-Pruis’s full name should be updated as stated above instead of “H. Voogdt-Pruis”.

Affiliation number 3. shoud read: “Department of Global Health, University Medical Centre Utrecht, Utrecht, the Netherlands” instead of “3EnCorps, Hilversum, the Netherlands”.

These errors have now been corrected online.

